# Protective effect of N-acetylcysteine on acute lung injury in septic rats by inhibiting inflammation, oxidation, and apoptosis

**DOI:** 10.22038/IJBMS.2022.65350.14384

**Published:** 2022-07

**Authors:** Jian-wei Le, Min Sun, Jian-hua Zhu, Heng Fan

**Affiliations:** 1 Department of Intensive Care Unit, Ningbo First Hospital, Ningbo, Zhejiang Province, P.R China

**Keywords:** Acute lung injury, Apoptosis, Inflammation, N-acetylcysteine, Oxidative stress, Sepsis

## Abstract

**Objective(s):**

Acute lung injury (ALI) is a common comorbidity in patients with sepsis, and finding drugs that can effectively reduce its mortality is a hot spot in current research. The purpose of this study is to explore the protective mechanism of N-acetylcysteine (NAC) on ALI in septic rats.

**Materials and Methods:**

We used NAC to intervene in septic rats to evaluate the plasma inflammatory factors and lung tissue pathological damage. Biochemical methods were used to determine the levels of oxidases in lung tissue, the expression of inducible nitric oxide synthase (iNOS) and endothelial nitric oxide synthase (eNOS) proteins, and observed lung tissue cell apoptosis.

**Results:**

NAC pretreatment decreased the mortality of septic rats, improved lung tissue pathological damage, reduced the levels of tumor necrosis factor-α, interleukin-1β, interleukin-6, interleukin-8, and malondialdehyde, and increased activity of superoxide dismutase, glutathione peroxidase, and catalase. Moreover, NAC pretreatment significantly decreased iNOS protein expression and increased eNOS protein expression in lung tissue. Meanwhile, NAC significantly decreased the number of apoptosis and the levels of Bax and Caspase-3 mRNA and increased the level of Bcl-2 mRNA in the lung tissue of septic rats.

**Conclusion:**

NAC protects ALI in septic rats by inhibiting inflammation, oxidative stress, and apoptosis.

## Introduction

Sepsis is one of the common complications after various surgical operations, hypoxia, reperfusion injury, severe burns, trauma, etc (1). It is also one of the main causes of death in intensive care units, and is due to the use of immunosuppressive agents and antibiotic resistance, making the prevalence of sepsis present an increasing trend year by year (2). The lung is often the first organ of failure in sepsis, and it has the characteristics of early-onset and high incidence. Due to endotoxin and other factors, inflammatory cells produce a large number of lipid metabolites and inflammatory mediators and stimulate inflammatory cells, especially macrophages and neutrophils to accumulate in the lung tissue, and the activation can aggravate the inflammatory response and produce a cascade joint reaction, inducing osmotic pulmonary edema and destruction of the air-blood barrier, resulting in the formation of hyaline membrane and the collapse of alveoli, which can cause pulmonary interstitial fibrosis in severe cases (3). 

Acute lung injury (ALI) is one of the main causes of death in patients with sepsis. Under the action of endotoxin and inflammatory chemokines, the inflammatory response of lung tissue is over-activated, leading to lung dysfunction, which in turn induces oxidative stress damage (4). Therefore, improving the inflammatory state in patients and preventing and treating ALI during the progression of sepsis is of great significance for improving the survival rate. N-acetylcysteine (NAC) has anti-oxidation, anti-inflammatory, anti-endotoxin, and other pharmacological effects, and is often used clinically for the treatment of diseases such as inflammation and bacterial infections (5). In recent years, studies confirmed that NAC has a significant therapeutic or ameliorating effect on ALI in rats caused by lipopolysaccharide (LPS), suggesting that NAC may have an ameliorating effect on ALI caused by sepsis (6).

In order to explore the protective effect of NAC on ALI in septic rats and its related mechanisms, in the present study, by replicating the rat model of sepsis, we used a variety of biomolecular methods to clarify the protective mechanism of NAC on ALI in septic rats and provided new ideas and references for the clinical treatment of patients with septic ALI.

## Materials and Methods

***Animals and grouping***

Male Sprague Dawley (SD) rats (Soochow University, Suzhou, China), weighing 350–450g, were randomly divided into 4 groups: Control group, Sham group, cecal ligation and puncture (CLP) group, and the CLP+NAC group, each group with 10 rats. Septic rat model preparation: after 12 hr of fasting, rats were anesthetized with pentobarbital (60 mg/kg), an incision was made (1.0 cm) in the middle of the abdomen, the peritoneum was cut, and the cecum was separated, the silk thread was ligated at 1/3 of the root of the cecum, and 3 places were perforated at the end of the cecum. The feces flowed out into the abdominal cavity, and the intestinal tube was returned to the abdominal cavity. The control group did not have any operation; in the Sham group only the abdomen was opened to separate the distal end of the cecum, without ligation and perforation, and the abdomen was closed directly. After the operation, the rats were kept in separate cages and were free to drink water. In the CLP+NAC group, NAC (2×10^4 ^U/kg, Sigma-Aldrich Co., Shanghai, China) was slowly injected into the tail vein 1 hr before CLP and repeated 24 hr later; the Sham and CLP group were injected with the same amount of saline solution at the above time points. Our study was approved by the Animal Ethics and Welfare Committee of Ningbo University (No. AEWC-2017-33).

***Hematoxylin-eosin (HE) staining***

The lower lobe of the left lung tissue was fixed in 10% formalin for HE staining. The changes in alveolar and pulmonary interstitial edema, infiltration of inflammatory cells, degree of pulmonary hemorrhage, and formation of pulmonary hyaline membrane were observed under a light microscope, and the lung tissues were scored pathologically. Scoring criteria: 0 points for no injury, 1 point for mild injury (<25%), 2 points for moderate injury (25%–50%), 3 points for severe injury (50% to 75%), 4 points for extremely severe injury (75%) (7). 

***Lung tissue wet/dry weight ratio***

We took the left upper lobe of the rat, washed it with 0.9% sodium chloride solution, and the filter paper was soaked dry and weighed as wet weight. We placed it in a constant 95 *°C* oven for 24 hr until the weight no longer changed to dry weight, and then calculated the lung tissue wet/dry weight ratio.

***Blood collection and testing***

After successful modeling, about 0.5 ml of blood was collected at 12 hr, 24 hr, and 48 hr, centrifuged at 3000 r/min for 10 min, and the supernatant was stored in a -80 °C refrigerator for later use. The concentrations of serum tumor necrosis factor-α (TNF-α), interleukin-1β(IL-1β), interleukin-6(IL-6), and interleukin-8 (IL-8) were detected in accordance with the enzyme-linked immunosorbent assay kit (ELISA, Jiancheng Co., Nanjing, China) instructions. 

***Detection of oxidative stress indicators***

The prepared lung tissue homogenate was centrifuged at 4 °C and then the supernatant was taken. The malondialdehyde (MDA, Jiancheng Co., Nanjing, China) level was determined by the thiobarbituric acid method, xanthine oxidase method to determine superoxide dismutase (SOD, Jiancheng Co, Nanjing, China) activity, potassium permanganate titration method to determine catalase (CAT, Jiancheng Co. Nanjing, China) activity, and biochemical analysis to detect glutathione peroxidase (GSH-Px, Jiancheng Co, Nanjing, China) activity. 

***Immunochemistry***

Twenty-four hours after modeling, lung tissue specimens were taken, paraffin sections were deparaffinized, hydrated, washed with phosphate-buffered saline, and sealed. 50 μl of anti-inducible nitric oxide synthase (iNOS, Boster Biotech Co., Wuhan, China) antibody and anti-endothelial nitric oxide synthase (eNOS, Boster Biotech Co., Wuhan, China) antibody were added to the slide tissue, and placed in a refrigerator at 4 *°C* overnight. Secondary antibody (Sigma-Aldrich Co., Shanghai, China) was dropwised and let stand at room temperature, streptavidin-biotin complex was dropwised, performed diaminobenzidine color staining, dehydrated, covered with neutral gum, and observed under a microscope. Three fields of view were selected in the positive expression area for each slice, and the cumulative optical density value of the immunohistochemical slice was detected by Image Pro Plus image analysis software (Media Cybernetics, Bethesda Co., USA), and the mean optical density was calculated. 

***TdT-mediated dUTP Nick-End Labeling (TUNEL)***

The specific steps followed the instructions of the kit (Sigma-Aldrich Co., Shanghai, China). Rats were sacrificed 48 hr after modeling. The lower lobe of left lung tissue was fixed in 10% formalin, dehydrated with ethanol, transparent with xylene, immersed in wax, embedded, and cut into 5 μm sections, and stained with the TUNEL method.

***RT-qPCR***

The mRNA was extracted from fresh lung tissue according to the operation steps in the instructions of the kit (Boshide Co., Wuhan, China). Using TaqMan Reverse Transcription Kit (ThermoFisher Co., Shanghai, China) reverse transcription, SYBR Green (ThermoFisher Co., Shanghai, China) method was used to detect qPCR. The detection primers are as follows: Caspase-3, Forward, 5’-CGG ATC CAT GGT CGC CTC CCG ATG TTT AC-3’; Reverse, 5’-CCT CGA GTC ATT AAA GTG ACT CCC AG-3’; Bax, Forward, 5’-AGC ACC AAC CCT CCC ATC ACC CTG CTC GC-3’; Reverse, 5’-GCA CCA GCC ACT ACA CAG CAT ACC CA-3’; Bcl-2, Forward, 5’-ACG CAC CTC GCA CGA CAT CC-3’; Reverse: 5’-ACG TAT GTT AGG CAG CAA CGT CTA TA-3’. The reaction system: cDNA, 5.0 μl; upstream primer, 0.5 μl; downstream primer, 0.5 μl; 2×SYBR Green PCR Master Mix, 10 μl, ddH_2_O, 4.0 μl, a total of 20 μl. The PCR amplification conditions were: 94 °C pre-denaturation for 5 min, then 94 °C for 30 sec, 55 °C for 30 sec, and 72 ° for 45 sec, 40 cycles. We used 2^-ΔΔCt^ to calculate the relative mRNA level, repeating 3 times for each sample. 

***Survival analysis***

SD rats were randomly divided into 6 groups: Control group, Sham group, CLP group, CLP+NAC(12.5mg/kg) group, CLP+NAC(25mg/kg) group, and the CLP+NAC(50mg/kg) group, each group with 20 rats. We observed the survival of rats in each group and recorded the survival status of rats within 7 days after modeling. 

***Statistical analysis***

SPSS Statistics 23.0 software (IBM Co, Hangzhou, China) was used to analyze the data, and multiple group comparisons were performed by one-way analysis of variance, pairwise comparisons were performed by LSD-t test, and survival analysis was performed by Log-rank. *P*<0.05 was defined as statistically significant.

## Results

***Protective effect of NAC on septic rats***

**Figure 1:**
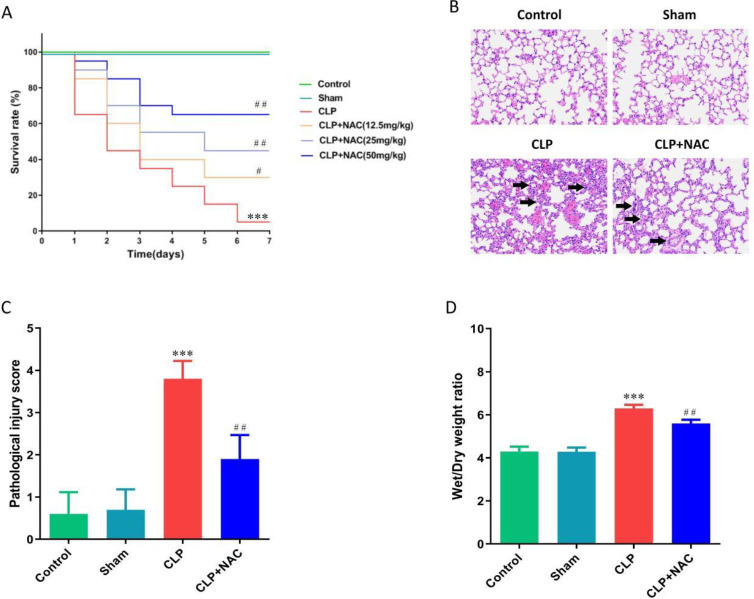
Effect of NAC pretreatment on mortality and lung pathological injury. (A) The survival rate of rats in different groups (n=20 in each group); (B) The pathological changes of lung tissue (HE, ×200); (C) Estimation of the lung injury score; (D) Lung tissue wet/dry weight ratio

NAC: N-acetylcysteine; CLP: cecal ligation and puncture Data are expressed as mean±SD. ***P<0.001 vs the Sham group; #*P*<0.05; ##*P*<0.01 vs the CLP group (n=10 in each group)

To determine the optimal therapeutic dose of NAC for septic rats, we observed the survival of the rats within 7 days. We found that the survival rate of rats in the CLP group was only 5% within 7 days, which was significantly lower than that in the Sham group (Log-rank=40.26). The survival rates of septic rats in different doses of the NAC intervention group within one week of modeling were 30% (NAC 12.5 mg/kg), 40% (NAC 25 mg/kg), and 50% (NAC 50 mg/kg). Among them, the 50 mg/kg NAC intervention improved the survival of septic rats to the greatest extent (Log-rank=14.78) (Figure 1A). Therefore, we used 50 mg/kg NAC for follow-up experiments. 

We further evaluated the lung injury of rats and calculated the lung injury score and wet/dry weight ratio. We found that the alveolar structure of the rats was clear in the Control and Sham groups. Both CLP and CLP+NAC groups had different degrees of lung injury. Among them, the CLP group had more severe lung injury, with inflammatory cell infiltration, alveolar edema, alveolar hyaline membrane formation, and thrombosis and atelectasis in the lungs. Compared with the CLP group, the pathological damage degree and wet/dry weight ratio in the CLP+NAC group were significantly reduced, the water content in the alveolar interstitium was reduced, the infiltration of inflammatory cells was reduced, the edema and bleeding were reduced, and the alveolar and tissue structures were clear (Figure 1B-D). It could be seen that 50 mg/kg NAC significantly improved the survival rate of septic rats and had a protective effect on ALI.

***The inflammation inhibitory effect of NAC on sepsis rats***

**Figure 2:**
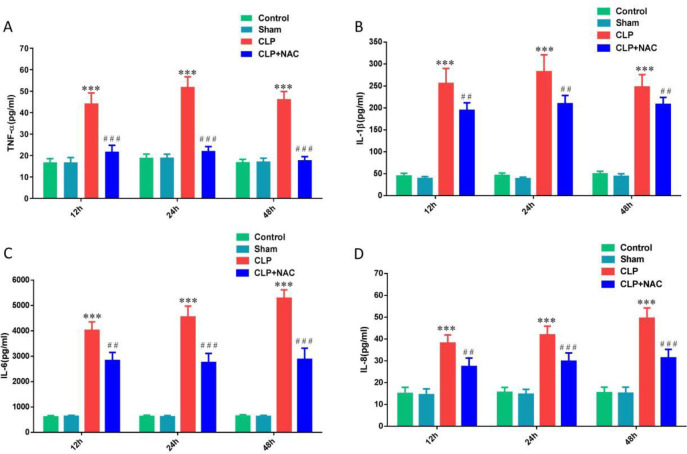
Effect of NAC pretreatment on inflammatory factors. (A) The level of TNF-α in serum; (B) The level of IL-1β in serum; (C) The level of IL-6 in serum; (D) The level of IL-8 in serum. NAC: N-acetylcysteine; TNF-α: tumor necrosis factor-α; IL-1β: interleukin 1β; IL-6: interleukin 6; IL-8: interleukin 8. Data are expressed as mean±SD. ***P<0.001 vs the Sham group; ##*P*<0.01; ###*P*<0.001 vs the CLP group (n=10 in each group)

To clarify the inflammation inhibitory effect of NAC on septic rats, we collected blood through the tail vein of the rats at 12 hr, 24 hr, and 48 hr and assessed the inflammatory factor levels. We found that the expressions of TNF-α, IL-1β, IL-6, and IL-8 were significantly increased in the CLP group. Among them, TNF-α and IL-1β were at the peak of inflammation at 24 hr. IL-6 and IL-8 were in the highest inflammatory response at 48 hr. NAC pretreatment significantly decreased the levels of TNF-α, IL-1β, IL-6, and IL-8 in the plasma of septic rats (Figure 2A-D). Our results showed that NAC could significantly inhibit the systemic inflammatory response of septic rats at different time points. 

***Anti-oxidant effect of NAC on ALI in septic rats***

**Figure 3:**
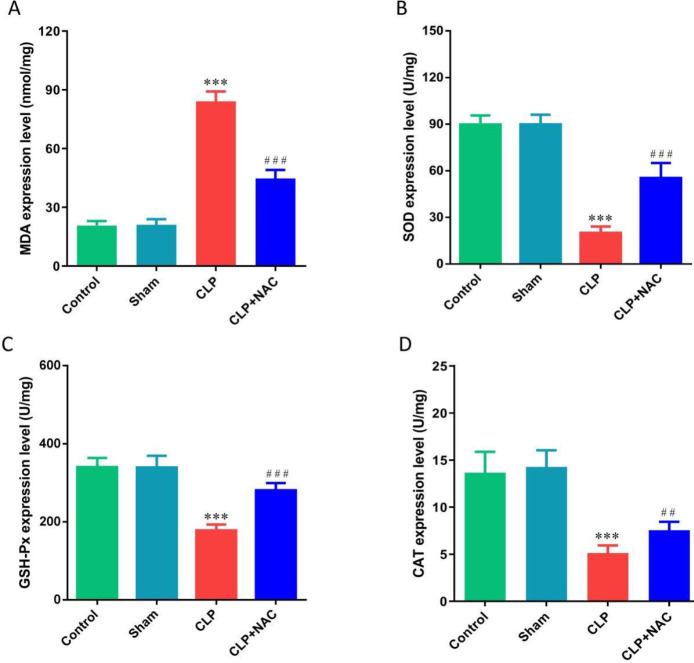
Effect of NAC pretreatment on the production of oxidase. (A) The levels of MDA in the lung tissue; (B) The levels of SOD in the lung tissue; (C) The levels of GSH-Px in the lung tissue; (D) The levels of CAT in the lung tissue

NAC: N-acetylcysteine; MDA: malondialdehyde; SOD: superoxide dismutase; GSH-Px: glutathione peroxidase; CAT: catalase

**Figure 4:**
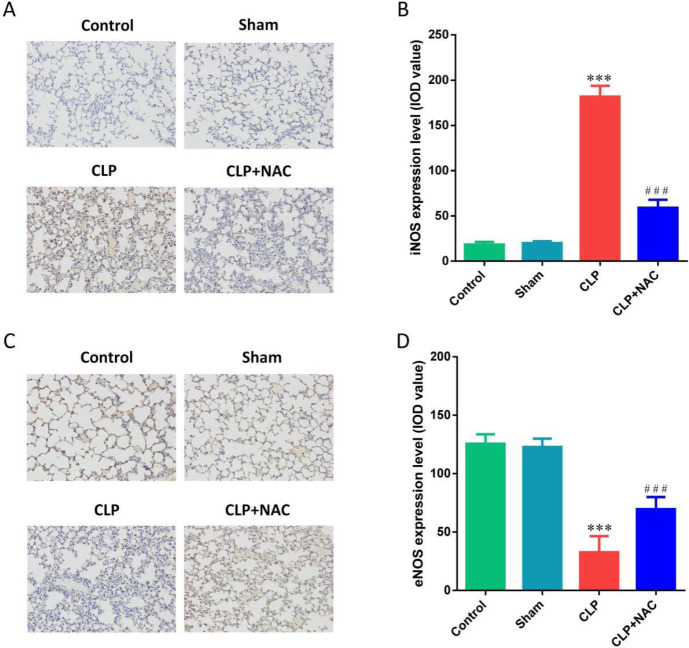
Effect of NAC pretreatment on the expressions of iNOS and eNOS. (A) The expressions of iNOS in the lung tissue (IHC, ×200); (B) The IOD value of iNOS in the lung tissue; (C) The expressions of eNOS in the lung tissue (IHC, ×200); (D) The IOD value of eNOS in the lung tissue

NAC: N-acetylcysteine; MDA: malondialdehyde; SOD: superoxide dismutase; GSH-Px: glutathione peroxidase; CAT: catalase; iNOS: inducible nitric oxide synthase; eNOS: endothelial nitric oxide synthase

To clarify the mechanism of NAC inhibiting oxidative stress in the lung tissue of septic rats, we measured the levels of MDA, SOD, GSH-Px, and CAT. We found that CLP induced a significant increase in the activity of MDA in the lung tissue of septic rats, and caused a significant decrease in the activities of SOD, GSH-Px, and CAT. NAC pretreatment significantly decreased the activity of MDA and increased the activities of SOD, GSH-Px, and CAT (Figures 3A-D). In addition, we also used immunohistochemistry to detect the expression of iNOS and eNOS in the lung tissue of rats. We found that CLP caused an increase in the expression of iNOS in the lung tissue of rats, and NAC pretreatment significantly reduced the expression of iNOS (Figures 4A, B). Contrary to the above results, CLP caused a decrease in the expression of eNOS in the lung tissue of rats, and NAC pretreatment significantly increased the expression of eNOS (Figures 4C, D). The above results confirmed that NAC protected ALI in septic rats with anti-oxidants.

***Anti-apoptotic effect of NAC on ALI in septic rats***

**Figure 5:**
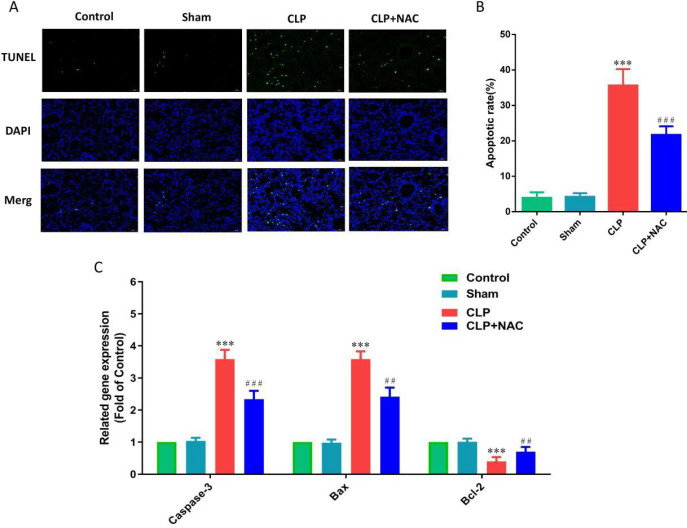
Effect of NAC pretreatment on lung tissue apoptosis. (A) The apoptotic cells of lung tissue (×400); (B) The apoptotic rate of lung tissue; (C) The mRNA levels of caspase-3, Bax, and Bcl-2

To study the anti-apoptotic effect of NAC on ALI in septic rats, We used TUNEL to evaluate the apoptosis of lung tissues in each group. We found that CLP induced apoptosis of a large number of alveolar epithelial cells in lung tissue, and NAC pretreatment significantly reversed this phenomenon (Figures 5A, B). In addition, we used RT-qPCR to detect the expression of apoptosis genes Caspase-3, Bcl-2, and Bax mRNA in the lung tissues. Our results showed that CLP induced an increase in the pro-apoptotic genes Caspase-3 and Bax mRNA in the lung tissue, and a decrease in the apoptosis-inhibiting gene Bcl-2 mRNA. Meanwhile, we found that NAC pretreatment significantly reduced Caspase-3 and Bax mRNA levels, and increased Bcl-2 mRNA levels (Figure 5C). It could be seen that NAC protected ALI in septic rats by inhibiting alveolar epithelial cell apoptosis. 

Data are expressed as mean±SD. ***P<0.001 vs the Sham group; ##*P*<0.01; ###*P*<0.001 vs the CLP group (n=10 in each group)

Data are expressed as mean±SD. ***P<0.001 vs the Sham group; ##*P*<0.01; ###*P*<0.001 vs the CLP group (n=10 in each group)

NAC: N-acetylcysteine

Data are expressed as mean±SD. ***P<0.001 vs the control group; ##*P*<0.01; ###*P*<0.001 vs the CLP group (n=10 in each group)

## Discussion

Sepsis is a common clinically severe disease, and multiple organ failure by inducing systemic inflammation is the main pathological feature (8). ALI is a serious complication that can occur in sepsis, with an incidence rate of 60~70%, and a fatality rate of 50%~70% (9). Therefore, protecting lung function and reducing acute injury are of great significance to improve the prognosis of sepsis. In this study, our results showed that after NAC pretreatment, the exudation of neutrophils was significantly reduced in septic rats, the degree of lung tissue damage was less, and the alveolar integrity was better. Our results showed that NAC could significantly reduce the mortality of septic rats and had a significant protective effect on ALI. 

LPS is the main “mediator” of sepsis leading to multiple organ complications, which can stimulate neutrophils and macrophages to release inflammatory cytokines (10, 11). In addition to direct damage to vascular endothelial cells of lung tissue, TNF-α can also stimulate the body’s immune stress by binding to the TNF-binding proteins of other inflammatory cells and further promote the production and release of inflammatory factors, leading to the occurrence of inflammatory cascades (12). IL-1β is a pro-inflammatory cytokine, which can also further stimulate immune stress and inflammation. IL-6 can cause release of a large amount of oxygen free radicals (ROS) (13). In the present study, our results indicated that NAC pretreatment effectively inhibited the increase of TNF-α, IL-1β, IL-6, and IL-8, and significantly inhibited the inflammatory response in the lung tissue of septic rats. 

The anti-oxidant enzymes SOD and CAT, which use ROS as substrates, are consumed in large quantities, and the activity of GSH-Px is reduced so that ROS cannot be cleared in time, which leads to oxidative stress damage to the lung tissue (14). In order to clarify the mechanism of NAC inhibiting oxidative stress in ALI of septic rats, we measured the levels of oxidases. We found that NAC pretreatment effectively inhibits the increase of MDA and also restores the activity of SOD, CAT, and GSH-Px in the lung tissues of septic rats, suggesting that NAC pretreatment inhibits the oxidative stress in the lung tissue. 

In the process of inflammation, nitric oxide (NO) plays an important role in the activation and migration of white blood cells and vasodilation (15). The cause may be closely related to vascular permeability, which can lead to abnormal alveolar-pulmonary capillary function (16). NAC is a commonly used anti-oxidant agent in clinical practice, which has multi-directional pharmacological effects, such as anti-inflammatory, anti-oxidant, and blood vessel protection (17). Our previous studies have found that an increase in the amount of NO can indicate lung tissue damage, and the increase in NO is often caused by the induction and activation of iNOS (18). The diversity of NOS expression in cells makes it in the process of lung injury occurrence and development. With complexity, the balance of iNOS and eNOS can reflect the degree of lung tissue damage. Our study found that NAC pretreatment could effectively inhibit iNOS expression and significantly promote eNOS expression, indicating that protection of NAC in lung tissue was closely related to its role in regulating the balance of iNOS/eNOS. 

Apoptosis is an important mechanism of sepsis, which directly affects the prognosis of patients with sepsis (19). Apoptosis is dominated by two pathways: intracellular and extracellular. The extracellular is related to the binding of TNF-α, Fas/FasL, tumor necrosis factor receptor, and Fas protein to activate caspase-8 (20, 21). It has been confirmed that TNF-α is one of the important inducers of cell apoptosis (22). Endogenous cells eliminate the mitochondrial membrane potential through the pro-apoptotic protein Bax, open the channel, release cytochrome c, activate caspase-9 under the action of apoptotic protein activator-1, and finally activate caspase-3 causing cell apoptosis (23). Our study showed that NAC could reduce the activation of Bax genes caused by the release of cytochrome C, and down-regulate the expression of apoptosis-related genes, thereby inhibiting cell apoptosis. 

## Conclusion

NAC can significantly reduce the mortality of septic rats and has a protective effect on ALI. The specific mechanism is closely related to the effect of NAC reducing the level of inflammatory factors, inhibiting oxidation, regulating the balance of iNOS/eNOS, and inhibiting apoptosis.

## Authors’ Contributions

HF Designed the experiments; JWL Performed experiments and collected data; JWL and MS discussed the results and strategy; JHZ Supervised, directed, and managed the study; JWL, MS, JHZ, and HF Approved the final version to be published.

## Conflicts of Interest

The authors declare that they have no conflicts of interest.
